# Perception of mental health in Pakistani nomads: An interpretative phenomenological analyses

**DOI:** 10.3402/qhw.v8i0.22469

**Published:** 2013-12-23

**Authors:** Fahad Riaz Choudhry, Iram Zehra Bokharey

**Affiliations:** 1 Clinical Psychologist, Centre for Clinical Psychology, University of the Punjab, Lahore, Pakistan; 2 Senior Clinical Psychologist, Services Hospital, Lahore, Pakistan

**Keywords:** Nomads, mental health, marginalized community

## Abstract

The study was conducted to explore the mental health issues of Pakistani nomads and to uncover their concept, ideation, and perception about mental health and illnesses. It was an exploratory study situated in the qualitative paradigm. The research strategy used was Interpretative Phenomenological Analysis (IPA), as the study was planned to explore the lived experiences of nomads regarding mental health and coping strategies and how they interpret those experiences. For data collection, focus group discussions (FGDs) were conducted. Seven participants were included in the FGDs, and two FGDs were conducted composed of both genders. The responses were recorded, and data were transcribed and analysed using IPA. Data verification procedures of peer review, which help to clarify researcher bias and rich thick description, were used. The major themes were lack of resources and myriad unfulfilled needs, specifically the basic needs (food, shelter, and drinking and bathing water). Moreover, a strong desire to fulfil the secondary needs of enjoyment and having luxuries was also reflected. A list of recommendations was forwarded for policy making of this marginalized community and to create awareness regarding mental health.

While travelling, irrespective of which country one is passing through, we often come across vacant lands where we see temporary tent houses in which a number of families are camped with animals and their carts around them. In European countries, some nomads possess caravans (mobile homes), but in most Asian countries, nomads use animals and carts as a means of transportation. In South East Asia, including Pakistan, it is a common sight to witness nomads in the majority of suburbs and villages. In Pakistan, they are found in all the four provinces, in northern and tribal areas, and also in the suburbs of the federal capital, Islamabad. Few among them travel with mobile games for children, and they search for festivals where poor families with their children can entertain themselves, which becomes a source for earning for nomads. Who are they? Where do they come from? Which is their native land? Where do they live? These are some queries that come into mind when we see these people. They are gypsies or nomads or pastoralists. They are a part of every land and yet a part of no land. They are present in almost every part of the world, but still they are considered as “the excluded ones.”

According to dictionary (*Oxford Dictionary*, 6th ed.), a *nomad* is a member of a tribe that moves with its animals from place to place. A literature review suggested a dearth of empirical evidence on the mental health of nomads in Pakistan. However, some studies have been conducted in different countries focussing on mental health and other areas of their lives. India alone is estimated to have a nomadic population of at least 60 million, that is, about 7–10% of the population (Krätli & Dyer, 2009).

In Afghanistan, 6% of the total population of the country is nomadic pastoralists. In Pakistan, no figures are available, but Pakistan does include significant concentrations of pastoralists, estimated to be in the millions, especially in Baluchistan and North West Frontier Province (Krätli & Dyer, 2009).

Cooper et al. (2006) conducted a qualitative study to clarify findings of the survey of the health status of gypsies and travellers by exploring their health-related beliefs and experiences. The homes or alternative community settings of the participants in five geographically dispersed study locations were selected in England. The participants consisted of 27 gypsies and travellers with an experience of ill health, purposively sampled from a large population. The results indicated that the experience of poor health and daily encounters of ill health among extended family members were normalized and accepted. The major themes related to health beliefs and the effects of lifestyle on health were as follows: the way of travelling, low expectations of health, self-reliance and staying in control, fatalism, and fear of death. In that group, ill health was seen as normal, an inevitable consequence of adverse social experiences, and is stoically and fatalistically accepted.

Parry et al. (2004) found in a study on gypsy travellers of the United Kingdom that they were twice as likely to be depressed and thrice more likely to be suffering from anxiety as compared to other populations. Moreover, the suicide rate among gypsies and nomads was found to be 8.5 times higher than in the general population of the United Kingdom (Harris & Barraclough, 1997).

A health needs assessment study by Cumbria NHS (2009) reported that 79% of gypsy travellers reported that either they themselves or their relatives suffered from depression, whereas the average prevalence of common mental health problems in England is 16.5% of the population.

Similarly, Twiselton & Huntington (2009) used mental health as a major theme during interviews and found that several nomads reported having chronic and severe mental health problems ranging from anxiety and depression to paranoia and symptoms of bipolar disorder.

Emadi et al. (1992) conducted an ethnographic study with nomadic pastoralists in Iran. The study was conducted with the objective of exploring the nomads’ views about their situation and the main problems they faced, and to improve government policies for supporting such communities. A participatory technique method was used, and research through action was done rather than relying on conventional approaches. Consequently, liaison meetings between nomads and governmental agencies were held, and these group discussions were analysed. It was concluded that there had been significant transformations in ways of thinking about the complex relationships between nomads in Iran and the environments in which they live and work. The nomadic pastoralists had been able to achieve some sort of “balance” between their environment and their economy through a long-time co-adaptation. However, this has changed over recent decades, as nomads are now being held liable for the significant degradation of the rangelands over which they migrate with their livestock. Efforts to improve the natural resource status of rangelands have traditionally been attempted through the use of technology transfer and centralized top-down planning (Emadi, 2004).

Sharma (2011) conducted a study on South Asian nomads. The aim of this study was to examine nomads’ access to education. The study was conducted after the “Right to Education Act” was passed in India in 2009 and some amendments in policy were done. Also, the geographical mapping was done for sampling purposes. This study aimed to assess the trends of education among nomads of South Asia and also the exploration of fulfilment of the need for education. The results revealed “that some nomads seek ways and means that might allow them to educate their children. Some among them have been led to seek an alternative out of pastoralism itself, and several nomadic groups have settled down near towns and villages with provisions in health and education.”

Jamadar (2012) conducted a quantitative study on the mental health of Indian nomads. The sample consisted of 300 nomads, out of which 150 males and 150 females from different places of Mysore District were administered a mental health inventory. The results revealed significant differences in the mental health of nomads. In all three subgroups of nomads (fortune tellers, cattlemen, and basketry), a high level of mental health was shown, and the fortune teller subgroup was found to have more mental health than the other two groups.

Although the concept of mental health tends to vary from individual to individual, there is usually some consensus among people living together in the form of a community.

Nomads seem to be a marginalized community around the globe and especially so in developing countries such as Pakistan. As a result, they have limited access to health facilities, particularly mental health services. Furthermore, mental health problems are usually attributed to supernatural reasons, such as possession, among uneducated individuals. Hence, the present study was conducted with the aim to explore mental health concepts of nomads, because the level of awareness and acceptance around these issues in this community seemed to be a worthwhile inquiry.

## Method

The present study was situated in the qualitative paradigm as it seemed to be best suited, considering the exploratory nature of the study. In order to explore nomads’ views and understanding of mental health, a detailed in-depth analysis was required that could only be done through qualitative research. Moreover, since nomads tend to be uneducated and generally are a closed group resisting any foreign intrusion, the qualitative approach could answer the inquiry question in a very comprehensive fashion.

### Interpretative phenomenological analysis

The strategy used in this study was Interpretative Phenomenological Analysis (IPA), as the aim was to explore nomads’ perceptions about mental health that they had formed on the basis of their experiences. IPA consists of a two-stage process, or a double hermeneutics, in which the participants are trying to make sense of their world and the researcher tries to make sense of their worldviews (Smith, 2007). Moreover, IPA offers a systematic approach towards the interpretation of first-hand accounts situated in their contexts (Smith, Flowers, & Larkin, 2009).

In the present study, our participants responded with a variety of explanations regarding mental health on the basis of their lived experiences. Later, we tried to give meaning to their responses during the process of analyses. In addition, we studied the perspectives of the participants using intersubjective inquiry (i.e., how they conceptualized mental health and how they interpreted their lived experiences regarding this concept).

### Data collection method

The most appropriate method for data collection in our study seemed to be focus group discussions (FGD) (Morgan, 1998); this is because nomads were a closed group and were hesitant to share their views in one-on-one interviews, so it was convenient for them to give responses openly in a group environment where they witnessed their peers doing the same (Morgan, 1998). Two FGDs were conducted on two different days (19 April 2012 and 22 April 2012) with the same participants. The duration of the first FGD was 120 min, and the second focus group, which was conducted after a gap of 2 days, lasted for 80 min as the saturation of themes was attained.

### Sample

Homogeneous purposive sampling (Creswell, 1998) was employed. Nomads tend to have homogeneous characteristics; therefore, this kind of purposive sampling was used.

An introduction to study objectives and a consent form were read aloud in the regional language (Punjabi), and their consent was taken. The willing participants were selected for the study, which comprised a total of seven (i.e., four males and three females).

### Inclusion criteria

Only those nomads who did not stay at one place for more than a period of 1 month were included in the study. Both male and female nomads were included. Those considering themselves nomads, or *khanabadosh* and *tapir vaas* in regional language, were selected. Adult nomads with the age of 18 years and older were included.

### Exclusion criteria

Peripatetic nomads were excluded as they mostly live in one place and travel only for the purpose of performing their tricks and games for children in places where festivals are arranged. Nomads living permanently on the sides of railway tracks as well as seminomads were also excluded.

The demographic characteristics of the participants are summarized in the following table:

**Table:** 

Name	Participant no.	Age	Gender	Marital status	Occupation	Caste	Districts of travelling
Dawood	P1	42	Male	Married	Selling goods and begging	Bhatti	Jhang, Toba Tek Singh, and Shorkot
Aslam	P2	35	Male	Married	*Phera* (selling goods)	Bhatti	Toba Tek Singh and Gojra
Babar	P3	32	Male	Married	Bike mechanic	Bhatti	Toba Tek Singh and Gojra
Saima	P4	24	Female	Single	Nomad/nil	Rajput	Toba Tek Singh
Amina	P5	55	Female	Widow	Begging	Rajput	Toba Tek Singh and Gojra
Nasreen	P6	40	Female	Married	Begging	Bhatti	Toba Tek Singh
Amir	P7	25	Male	Single	Begging	Rajput	Toba Tek Singh

### Procedure and setting

The data collection proved to be a rather tedious and challenging task. Nomads turned out to be a very resistant and even aggressive group; therefore, it seemed almost impossible to get down to the actual study right away. Thus, it was decided to carry out a few pilot studies before carrying out the actual study. The pilot studies were conducted in the suburbs of Lahore and Toba Tek Singh, whereas the actual study was carried out in the suburbs of Toba Tek Singh. The main inquiry question was “What is nomads’ opinion about mental health issues?” We had also developed subquestions that served as an aid to answer this question in a comprehensive fashion. During FGDs, responses of the participants were audio recorded using a digital recorder mp4 device. The recording device was checked in advance before going to the field, by recording voices in an open environment. An audio tape recorder was also taken to the field as a backup. Recorded data was transcribed into text in Roman-Punjabi scripts (i.e.Punjabi language typed in English alphabets). Once the data were transcribed, multiple passes through the data were made, and significant themes identified. Thereafter, the similarities and differences in the themes were identified, and clusters of subthemes formed. The analyses were verified through peer review (Creswell, 1998), clarifying researcher bias and the soundness of the research (Marshall & Rossman, 2011).

### Pilot study

In the very first attempt at a pilot study, we tried interacting with nomads camped on vacant land near the graveyard of a village. We had not prepared FGD questions by then, but we introduced ourselves as students and told them the aim of this study and that we wanted to take their views regarding mental health. A middle-aged nomad whispered something and talked to the group members in a dialect of Punjabi language that was a bit difficult to comprehend. After that, all of them refused to talk and only responded with one common answer: that they were not nomads, they lived in the city of Toba Tek Singh, and they just happened to be passing by that place. Despite our repeated attempts to persuade them to cooperate, they did not give in and after a few minutes stopped listening to us.

In another attempt, we conducted a pilot test of FGD questions before conducting the planned study. For the pilot study, we visited a suburban area in the surroundings of Mughalpura, Lahore, where we found some peripatetic nomads, who are the typical entertainers for children, but they refused to give consent for the study. Then, we were told by a friend that we could find nomads beside the railway lines. Therefore, accompanied by our friend, we travelled in search of them side by side with the railway track and found camps of nomads there. They were semi-nomads and gave consent for the FGD. A group with five participants was conducted, and the questions developed for FGDs were asked and practised. The responses of one old participant gave us insight to rephrase a question because he reacted to the word *pagalpan* (i.e., madness). A strong denial and unwelcoming response were given by the group after the angry response of the old man. After that, we prepared some probes for each question of the FGD. The old participant became reactive, did not allow others to respond and continue the group, and expressed harsh words. He considered all people insane who were settled and not living a nomadic life. He also tried hitting us with a stick and wanted us to immediately quit questioning. The group was then continued in another adjacent camp, and some people controlled that old man from further interference. In the second attempt at a pilot study, we visited the nomads within the city of Lahore and to our dismay encountered yet another resistant and non-cooperative group. The male nomads refused to give their views and suggested their females to answer our questions. Two female nomads were present in the tent, and they shared their views, which were quite similar to the ones given by females in the actual study (i.e., they talked about rejection and helplessness, and denied the presence of mental illness in their community).

### The actual study

The challenges faced in the pilot study guided us to employ a gatekeeper to conduct the actual study. The gatekeeper was a female health worker who lived in Toba Tek Singh and had been interacting with nomads in that area for the past 15 years regarding various health-related campaigns (e.g., polio and family planning). We visited villages around Toba Tek Singh where our research gatekeeper accompanied us to the different slums of nomads. It took 2 complete days to find our target population as the majority of nomads who we found did not fulfil the inclusion criterion of frequent travelling. Finally, the health worker and local friends of that area identified the kind of nomads for whom we were searching, and after fulfilling the ethical requirements we conducted the FGDs.

### Ethical considerations

This project was scrutinized and approved by the Departmental Doctoral Programme Committee (DDPC) at the Centre for Clinical Psychology, University of the Punjab, Lahore, Pakistan. However, in Pakistan the ethical approval of a government department is not required to do any research on nomads as they are not duly placed under the management of any provincial or federal institution.

We met our participant group for the first time in the evening around 6 p.m. We introduced ourselves, and informal interaction was started as a part of rapport building. They were informed about this study. They asked the gatekeeper to take permission from their *maalik*, the owner of the land where they had placed their camps at that time. The owners of the land were contacted the next day, and they said they also wanted to be present during the FGDs. A rapport-building session was done on the second day, and the group was identified by giving them the open choice that we needed 6 to 12 individuals for the study. Eight participants gave their consent verbally. We arranged tea, biscuits, and some sweets for their children in order to facilitate a congenial working relationship with them. On the third day, initially the owners of the land were briefed about our study, and they advised the nomads to cooperate with us and left the place. Informed consent was taken from the participants by reading the consent form loudly and repeatedly in Punjabi, and seven participants gave written consent. They were briefed about the aims of the study. They were informed about their right to withdraw their participation at any stage of the research. Confidentiality was assured to them, and they were told that their personal information would be kept secret and that their responses would not be published with their names. Monitory compensation was given to the participants for giving their time and input. Each participant was paid Rs.300 for his or her participation. They were told that, if needed, free counselling services would be provided at the Department of Psychiatry Services Hospital in Lahore and at the Centre for Clinical Psychology at the University of the Punjab, Lahore. I gave them the address and contact number of these two places as well as my personal contact number. Consent for audio recording of the FGDs was also taken from them. Moreover, consent for taking and publishing their photographs was taken in the form of verbal and written record.

## Findings and discussion

### Concept of mental illness and mental health

This subsection addresses the concept of the participants regarding mental health and various issues related to it. Initially, a strong denial was witnessed among the participants as they said that they had never come across any mental health problem in their community. Interestingly, the denial seemed to exist on a collective level.

### Denial

The most common theme extracted from the responses was denial. Participants were asked about their concepts of mental health and illness, and, rather than responding to the characteristics, they started denying and clarified that such mental health issues were not present in their group, even though during the FGDs, a 10-year-old female child was roaming around who seemed to have mental retardation. Thus, as reported by Mr Aslam:

Our mental health is intact and none of us suffers from any mental health problem.

Similarly, Ms Saima said. “We are perfectly normal and none of us suffers from such mental health issues.”

According to Freud (1936; also see Moore & Rubinfine, 1969), denial is the strategy that the mind uses not to pay any attention to a given reality. An important point worth mentioning is that there are few people whose denial is based on their inability to face reality, whereas others may have the ability to understand reality but avoid doing so to avoid a conflict.

Blackman (2004) classified denial into four types. The first type is *denial per se*, which is the kind of denial in which reality is rejected despite the presence of overwhelming evidence. The second type is *denial in deed*, which is manifested through behaviour that symbolically states that a nasty reality is untrue (e.g., in the present study, the behaviour of the 10-year-old child was inappropriate and was depicting abnormality, but the nomads considered her absolutely fine and linked it with spiritual phenomena). The third type is *denial in fantasy*, which comprises the maintenance of erroneous beliefs to ignore facing a harsh reality. The fourth and final type is called *denial by words*, which involves the use of magical words to convince that a presented reality is false (Blackman, 2004).

All of these kinds of denial were manifested strongly through the responses of the participants of this study. Denial responses in regard to the possibility of having poor mental health in their community were revealed from the majority of the responses.

If we consider the sociocultural aspect of denial, it is quite obvious in our society that mental health problems are often considered a source of embarrassment, and people tend to deny them because of the stigma attached to them. In addition, the denial might also be due to lack of awareness concerning mental health issues, especially among those in the lower socioeconomic class such as nomads.

### Indicators of poor or stable mental health

The parameters of stable and poor mental health were clearly stated by nomads. Firstly, they expressed their views regarding poor mental health. For example, Ms Saima stated, “We consider a person insane who shows inappropriate behaviour, has poor self-care and does not wear proper clothes and footwear.” This seems to be an adequate *behavioural description* of poor mental health.

Secondly, they provided the disease model of poor mental health (i.e., it is a disease). Similarly, while expressing their views regarding stable mental health, they focussed on health, freshness, and fulfilment of one's basic needs. They believed that mental problems could be due to medical ailment or spiritual disease. By spiritual disease, they meant the occult, black magic, and the powers of amulets. Such views are quite common in our part of the world, especially among underprivileged and uneducated groups, who often derive solace and perspective from these beliefs.

Srole and Langner (1962) conducted a detailed analysis on religions and mental health affiliations. The results have shown that “religious origin” was the main source in the formation of the symptoms and the approach regarding mental health professionals. Other empirical studies (Cohen et al., 2012; Cornah, 2006) have also demonstrated the relationship of religiosity and mental health.

As a sociocultural aspect of our society, strong beliefs in the existence of occult and black magic, its practice, and consulting magic practitioners and faith healers are common, especially in the middle and lower socioeconomic classes. Belief in magic is further strengthened as it is also cited in the sacred book, the Qur'an. This might be the reason why the nomads also believed strongly in magic, and they attributed the mental health issues to parapsychological and magical phenomena. According to The Holy Qur'an Surah al Baqarah, verse 102:

They followed what the Shayatin (devils) gave out (falsely of the magic) in the lifetime of Sulayman, Alayhi Salaam (Solomon). Sulayman Alayhialaam did not disbelieve, but the Shayatin (devils) disbelieved, teaching men magic and such things that came down at Babylon to the two angels, Harut and Marut, but neither of these two (angels) taught anyone (such things) till they had said, “We are only for trial, so disbelieve not (by learning this magic from us).” And from these (angels) people learn that by which they cause separation between man and his wife, but they could not thus harm anyone except by Allah's Leave. And they learn that which harms them and profits them not. And indeed they knew that the buyers of it (magic) would have no share in the Hereafter. And how bad indeed was that for which they sold their own selves, if they but knew.

However, considering their lack of awareness and access to education, their unfulfilled basic needs, and the rejection they faced from society, it can be concluded that this might be their escape to avoid the stressful conditions of mental health problems; hence, they attributed such problems to witchcraft and magic.

### Causes of mental illnesses

The major factors that they considered responsible for poor mental health were unfulfilled needs and poverty. They discussed at length their myriads of unfulfilled basic and secondary needs.

### Need fulfilment

Nomads reported their deplorable living conditions and explicitly expressed their unfulfilled basic needs. They did not even have access to drinking or bathing water. As Ms Amina reported, “We do not have access to water and we are left with no other choice than drinking contaminated water and have no water for bathing.”

Mr Babar shared, “We have no shelters; even our mothers and sisters have no place and water for bathing.”

They expressed the desire of getting education and orientation about proper religious values for their next generation. They didn't have land for shelter and were so economically underprivileged that they had to continue their lifestyle of begging and moving. As shared by Mr Amir, “We want our children to get education and opportunities to learn religious knowledge and to have proper homes so that we can end our wandering life and stop begging.” The female participants highlighted the secondary needs issue. As reported by Saima and endorsed by other females, “We also want to do shopping, go for outing and enjoyments like other females of society.”

Self-determination theory (Deci et al., 2001) explains that individuals’ needs to experience autonomy and feel competence and relatedness are the core elements for growth and satisfaction. One's wellbeing, satisfaction, as well as resilience improve with the fulfilment of these three needs. In the context of nomads, it was observed that they experienced autonomy as they were free souls moving from place to place and had detached themselves from society. They seemed to have strong interpersonal relationships, as no theme appeared regarding conflicts of relationships. However, these findings go against the first part of this theory, as nomads did not report being satisfied on account of their poverty and the uncertainty element in their lives (i.e., they did not stay at one place and had continuous stress from travelling and adjusting to temporary places). The rejection that they faced from society might be another reason that triggered their stress. However, the second part of the theory, which highlighted the resilience enhancement, is well related to the situation of the nomads of our study (Deci et al., 2001)

Several studies show that need fulfilment is indeed related to subjective wellbeing. According to Deci, Ryan, Gagne, Leone, Usunov, and Kornazheva (2001), need satisfaction was increased when autonomy was granted. Furthermore, this need satisfaction was related to wellbeing. According to Prooijen (2009), when need for autonomy is not fulfilled, people become more sensitive towards justice and fairness procedures. Experimental studies have validated these results. Sheldon and Filak (2008) manipulated relationship, competence, and autonomy need in an experiment. These results revealed that intrinsic motivation and mood were affected by these manipulations. Participants of this study had their need of autonomy fulfilled, but unlike the studies reported above, they were not satisfied, and this could have led to their angry and sad mood.

### Management of mental health issues

As mentioned above, two perspectives emerged in the definition of mental health: it was either a spiritual disease or a medical one. It was interesting to note that the same two treatment options were outlined by nomads for mental health problems.

Ms Nasreen reported, “We visit our faith healer saint Haider Shah whenever faced with any mental health or other problems and through his prayers and blessings God cures the disease.”

However, Mr Dawood and Mr Aslam said, “If we have any health or mental health problem we consult medical doctors in the nearby dispensary.”

They expressed that when they came across any mental health problem, they consulted a faith healer or a doctor; however, their preference was to consult a faith healer. Few believed that such people with mental health issues were not suffering from any disease; rather, they had some spiritual connections with God and were the chosen ones. The prevalent belief was that by visiting shrines, the mentally ill individuals could be healed.

Ms Saima reported, “This is not abnormality; such people are ‘Allah Log’ (God's special people); they eat holy meal at shrines and are a manifestation of God's will.”

In Pakistani society, this dilemma of making a choice between a psychological or medical problem and a spiritual phenomenon is very common. This belief is found not only in lower class segments of society but also in middle-class and some wealthy ones, as they prefer visiting shrines and consulting faith healers in order to get treatment for psychiatric disorders. Nomads’ views in this regard were not surprising as they had learned them from local subcultures (i.e., suburbs and villages—areas that are known for shrines and spiritual people famous for amulets and treating such patients).

### Response to stress

Female nomads expressed that they faced stressful situations during social interactions. When people rejected them due to their poverty and bad odour, it aggravated their stress level. Ms Nasreen reported, “People consider us mad because of poor hygiene, bad odour and poverty … this is because of lack of money. If we have resources for bathing and dressing then people give respect and offer us to sit with them.”

Other participants also considered their poverty and the arrangement of twice-daily meals as the most stressful aspect of their lives.

The cognitive-relational theory of Lazarus conceptualized stress as “a meticulous relationship between environment and individual that is determined by the person as moving or passing ahead of his resources and putting his wellbeing in danger” (Lazarus & Folkman, 1984, p. 19). Demands of environment and personal resources are perceived at the same time to establish appraisals. However, altered requirements and improvement in personal abilities are a few parameters that can change appraisals with time.

The stress cognitive-relational theory draws attention towards the person and the environment, their interaction, and the nonstop reciprocal relationship between them (Lazarus & Folkman, 1984).

Experiences of stress and results of coping bring together direct instant effects, like physiological changes (i.e., increased or decreased sleep), and lasting consequences regarding issues of psychological well-being and somatic and social functioning. Nomads reported that they often resorted to sleep in the face of stress. Furthermore, they often indulged in crying and praying as a way to cope with their stress. Mr Amir stated, “What can we do? We just go to sleep and pray to God.” This is supported by the theory put forward by Coifman et al. (2004), which says that the flexible use of emotions can result in decreased distress. Similarly, nomads used their emotions in a flexible fashion (American Psychiatric Association, 2000). However, Ms Nasreen gave a somewhat different response from the others when she said:

To overcome stress we wash our clothes, freshen ourselves and go for outing, people accept us and we feel better.

The present study was perhaps one of the few attempts in Pakistan to address the concept of mental health amongst nomads. The insights drawn from this study point towards a need to carry out empirical research exploring multiple dimensions of their lives so as to enable the policy makers to help address their concerns.

**Figure:**
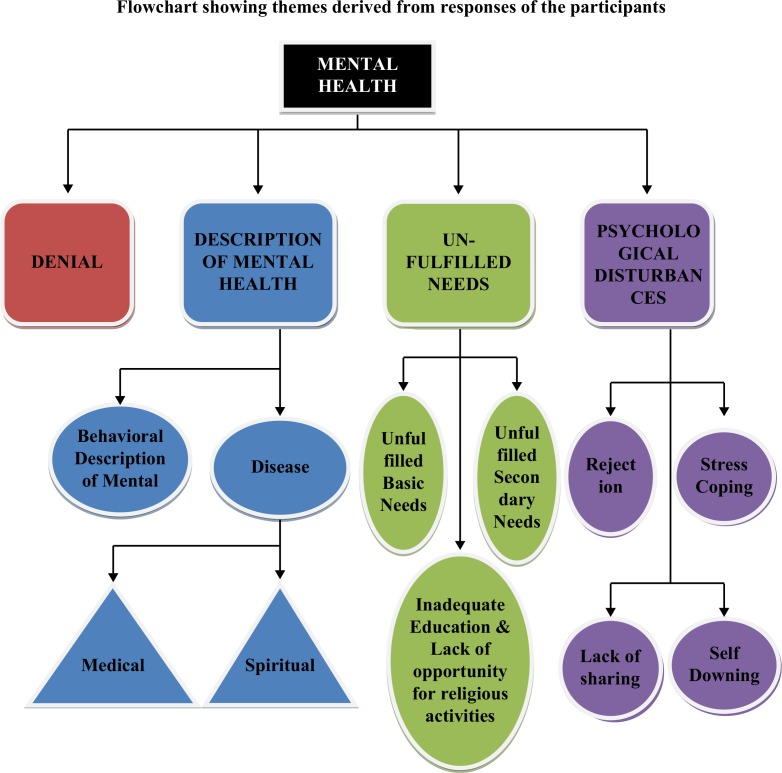


## Limitations

Some problems faced in the study need to be mentioned. First and foremost, mapping and identifying the “nomad-prone slums” were quite difficult tasks, as we consulted many people in different villages who guided us regarding nomads’ camping sites. Moreover, travelling in order to find and approach the participants was the most challenging and time-consuming part of the study, as nomads are “free souls” and excluded ones from society, and they do not come under any governmental authority. Surprisingly, they are not even registered as citizens of the country, and, consequently, the majority of them did not have National Identity Cards. Some other method of distributing monetary compensation should have been adopted, as a mob of nomads attacked us while we were distributing money. Lastly, language limitations also need to be acknowledged. It took us some time to understand their colloquial dialect (Punjabi), and the pilot studies proved helpful in this regard.

## Implications and suggestions

This study points towards devising policies to recognize nomads as the citizens of the country in which they live, and make consolidated efforts to fulfil their needs and rights as proposed by the Dana Declaration (2002) and recommended by the International Labour Organization (1989) and the UN Declaration (2006) on the Rights of Mobile Indigenous People. Moreover, efforts should also be made to equip nomads with educational and vocational skills in order to fulfil their basic needs and enhance their self-esteem. Last but not least, outreach programs to detect and treat mental health problems in them should also be developed UN Declaration (2006).
